# From pixels to isolation: Exploring the relationship between technological addiction, depression, hikikomori risk factors, and hikikomori tendencies among young adults

**DOI:** 10.1177/00207640251347470

**Published:** 2025-06-25

**Authors:** Patrick K. F. Lin, Philmon K. H. Lim, Yong Jie Yow, Jing Lee, Yui Annabelle Akiyama

**Affiliations:** James Cook University - Singapore Campus, Singapore

**Keywords:** Internet addiction, smartphone addiction, online gaming addiction, depression, Hikikomori risk factors, Hikikomori tendencies

## Abstract

The present study examined the relationships between technological addiction, depression, and Hikikomori tendencies, aiming to establish a directional model that explores their connections with Hikikomori risk factors. In two studies (Study 1: cross-sectional, *N* = 182; Study 2: quasi-experimental, *N* = 141), we found that technological addiction, depression, and Hikikomori risk factors were positively correlated with Hikikomori tendencies among young adults. Crucially, the impact of technological addiction on Hikikomori tendencies was mediated by depression and Hikikomori risk factors. The results suggest that technological addiction may contribute to increased depression among young adults, which in turn leads to Hikikomori-related risk behaviors (e.g., avoiding school or work) and ultimately results in the development of Hikikomori. Recommendations for interventions to mitigate Hikikomori tendencies and the influence of demographic factors (e.g., gender and geographical location) on Hikikomori are also addressed in the discussion section.

Hikikomori is a sociocultural and mental health phenomenon involving severe social withdrawal, primarily affecting young individuals. It causes significant distress to affected individuals and their caregivers (Saito, 1998). Although the actual causes remain under investigation, some studies suggest that technological addiction and depression may contribute to Hikikomori tendencies (e.g., Aguglia et al., 2010; Koyama et al. 2010; Stip et al., 2016; Tang & Koh, 2017). Due to the complex interrelationships between technological addiction, depression, and Hikikomori tendencies, most research has focused on one of the bidirectional influences: (a) between technological addiction and depression (e.g., Xie et al., 2023), (b) between technological addiction and Hikikomori tendencies (e.g., Amendola et al., 2023), or (c) between depression and Hikikomori tendencies (e.g., Teo, 2013). Acknowledging the cyclical nature of these relationships, the present research aims to explore the relationships between technological addiction, depression, and Hikikomori tendencies. Specifically, we seek to establish a directional model that elucidates the connections among these variables, with the addition of Hikikomori risk factors (e.g., social isolation, employment, and educational disengagement).

## Relationship between technological addiction, depression, and Hikikomori tendencies among young adults

The advent of the internet has transformed communication methods, especially through social networking sites (SNS), which allow individuals to connect with others and establish virtual environments (Kaplan & Haenlein, 2010; Vahedi & Zannella, 2021). Individuals can access various SNS via different technologies, such as computers, smartphones, and gaming consoles, making them ubiquitous in modern society. While technological advancements facilitate virtual connections, the widespread adoption of these technologies also increases the risk of technological addiction (Ko et al., 2012). Studies have shown that since the COVID-19 pandemic, approximately a quarter of the global population has been affected by some form of technological addiction (e.g., Onukwuli et al., 2023; Siste et al., 2021). Notably, the prevalence of technological addiction is especially high among young adults.

For instance, a study by Tang and Koh (2017) found that 29.5% of 1,110 college students in Singapore developed some form of SNS addiction. Similar patterns of SNS addiction have been observed in other parts of the world, with young adults experiencing technological addiction related to internet use, online gaming, and excessive smartphone use (e.g., Pham et al., 2025; Tang et al., 2017). Excessive use of SNS can lead to withdrawal symptoms, which in turn result in negative psychopathological and psychosocial outcomes (Skues et al., 2016; Tan & Chew, 2024). Some studies have shown that SNS addiction often co-occurs with depression (e.g., Tang & Koh, 2017), while others indicate that young adults with technological addiction tend to socially withdraw themselves (Amendola et al., 2023).

### Technological addiction on depression

Research has shown that young adults are susceptible to technological addiction. Several research reported that young adults who are afflicted with internet addiction (IA; Tang & Koh, 2017; Xie et al., 2023), smartphone addiction (SPA; Gümüştakim et al., 2024; Pham et al., 2025), and internet gaming disorder (IGD; Chew et al., 2024; Király et al., 2018) are also prone to depression (e.g., Elhai et al., 2017; Fumero et al., 2018; Ye et al., 2023). A study by Karakose et al. (2023) has shown the growing trend between technological addiction and depression. They analyzed three periods (i.e., 1983–2016; 2017–2019; 2020–2023) – there were increasingly more reported cases and studies on technological addiction and depression across each of the time periods respectively.

### Technological addiction on Hikikomori tendencies

While research on the link between technological addiction and depression is abundant, there are fewer studies on the relationship between technological addiction and Hikikomori tendencies (Kubo et al., 2022). In recent years, research from East Asia has shown that an increase in technological usage would increase hikikomori tendencies. For example, Tateno et al. (2019) found that increased usage of the internet, online gaming, and smartphones among Japanese college and university students led to isolation from social communities. In South Korea, 56% of Hikikomori young adults were at risk of internet addiction (Stip et al., 2016). Though most research examined young adults in East Asia, studies have also shown significant association between internet and gaming addiction on social isolation in Indonesia (Sujarwoto et al., 2023), India (Solanki et al., 2023), Italy (Orsolini et al., 2022), and the USA (Tran & Kay, 2024.) Hence, there is a strong possible link between excessive technology use and higher Hikikomori tendencies among young adults.

### Depression and Hikikomori tendencies

Research consistently demonstrates a strong link between depression and Hikikomori tendencies. In a case study, Teo (2013) found that his subject who was socially isolated also experienced severe depressive episodes. This pattern aligned with studies from China, Italy, Japan, Korea, and Spain, which show that increased loneliness correlated with a higher risk of depression (e.g., Jeong et al., 2021; Liao et al., 2020; Teo et al., 2014; Orsolini et al., 2023). Recent research has further solidified the connection between depression and Hikikomori. For example, Castelpietra et al. (2021) suggested that individuals with a history of Hikikomori are six times more likely to have a mood disorder compared to the general population. While some researchers have proposed that Hikikomori might be a variant of known mental disorders such as social anxiety, avoidant personality disorder, or adjustment disorder (e.g., Amendola, 2024), the prevailing view in current research maintains that Hikikomori is closely associated with major depressive disorders (e.g., Lee et al., 2022).

### Other factors relating to Hikikomori tendencies

While the present study focuses on the relationship between technological addiction, depression, and Hikikomori tendencies, prior research has identified additional contributing factors. Psychosocial influences such as traumatic childhood events, inadequate social support, gender expectations, and Hikikomori risk factors (e.g., unstable employment, school refusal, and low socioeconomic status) may exacerbate Hikikomori tendencies (Frankova, 2019; Liew et al., 2021). Recent studies have shown that these risk factors could be one of the potential antecedents linking to the onset of social withdrawal tendencies (Lin et al., 2022; Uchida & Norasakkunkit, 2015).

## The present research

While the relationships between technological addiction, depression, and Hikikomori tendencies are widely acknowledged as interconnected, our research posits a directional model linking these variables. To present more nuanced results, we investigated technological addiction as three separate constructs – namely internet addiction, smartphone addiction, and internet gaming disorder. We conducted two studies to investigate these dynamics.

**Figure 1.**
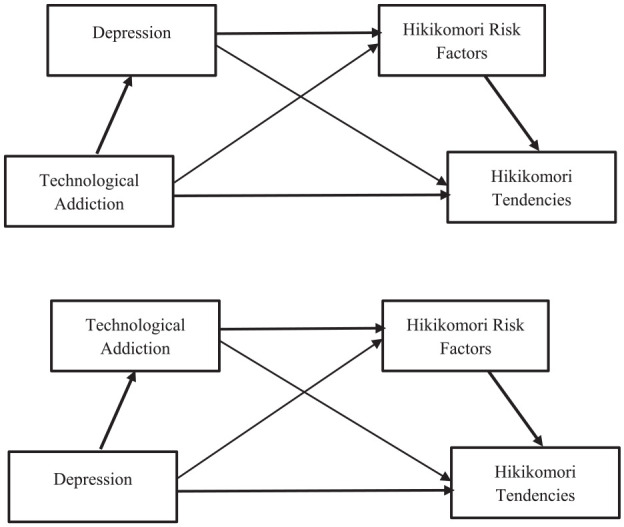
Proposed mediational models. *Note.* Pathway 1 – Technological addiction → Depression → Hikikomori risk factors → Hikikomori tendencies Link. Pathway 2 – Depression → Technological addiction → Hikikomori risk factors *→* Hikikomori tendencies Link.

Study 1 aimed to: (a) Examine correlations among technological addiction (i.e., internet addiction, smartphone addiction, and internet gaming addiction), depression, Hikikomori risk factors, and Hikikomori tendencies and (b) Test the possible mediational models between these variables. We proposed two possible pathways, as presented in Figure 1.

Pathway l: Technological addiction affects depression which in turn activates Hikikomori risk factors, leading to Hikikomori tendencies.
Pathway 2: Depression impacts technological addiction which leads to higher Hikikomori risk and then greater Hikikomori tendencies.

To enable stronger causal claims regarding the mediation model, (c) we employed a quasi-experimental design and examined the impact of technological addiction on Hikikomori tendencies with depression and Hikikomori risk factors as the mediators in Study 2.

## Study 1 – method

### Participants and design

Using a with medium effect size (f^2^ = .15), power = .85, and alpha .05, an a-priori Gpower analysis indicated that we required 102 participants. Accordingly, we recruited 182 undergraduates (*M* = 22.13, *SD* = 3.76; 49 males, 128 females, 5 others) via university research participation pool and social media platforms (e.g., Telegram, WhatsApp) for Study 1. The majority of participants were from Southeast Asia (69.18%), other participants were from other part of Asia. A cross-sectional design was used to examine the relationships between technological addiction, depression, Hikikomori risk factors, and Hikikomori tendencies.

### Materials

#### Technological addiction

##### Internet addiction test

The Internet Addiction Test (IAT; Young, 2009) is a self-reported questionnaire consisting of 20 items (e.g., *How often do you lose sleep due to being online?*), rated on a 6-point Likert scale (0 = Not Applicable; 5 = Always) to measure the severity of IA and its impact on individual’s social interactions, work, emotional stability, and daily functioning. Scores on the IAT is calculated by summing the responses of the 20 items, with higher scores indicating a greater possibility of IA. The Cronbach’s α for the IAT in the present study was .93.

##### Smartphone Addiction Scale Short Version

The Smartphone Addiction Scale Short Version (SAS-SV; Kwon et al., 2013) was designed to assess smartphone addiction in both clinical and research settings. The SAS-SV is a self-reported measure with 10 items (e.g., *Using my smartphone longer than I had intended*) on a 6-point Likert scale (1 = strongly disagree; 6 = strongly agree). The SAS-SV covers a range of behaviors and emotions associated with problematic smartphone use. The score is based on the sum of all SAS-SV items. The Cronbach’s α for the SAS-SV was .91.

##### Internet Gaming Disorder Scale Short Form

The Internet Gaming Disorder Scale-Short-Form (IGDS9-SF; Pontes & Griffiths, 2015) is a self-reported 9 items (e.g., *Do you feel more irritability, anxiety or even sadness when you try to either reduce or stop your gaming activity?*) scale to measure the severity of internet gaming disorder based on the criteria highlighted in the DSM-5. It is also used to examine problematic gaming. The IGDS9-SF score is based on the sum of 9 items on a 5-point Likert scale (1 = Never; 5 = Very Often). The Cronbach’s α for the IGDS9-SF was .90.

#### Depression

The score for depression was measured using the Depression Anxiety and Stress Scales-21 (DASS-21; Lovibond & Lovibond, 1995). Scores for the depression subscale were computed by summing the seven items that measured depression (e.g., *I couldn’t seem to experience any positive feeling at all*), and multiplied by 2.Items are rated on a 4-point Likert scale (0 = Did not apply to me at all; 3 = Applied to me very much, or most of the time). The Cronbach’s α for the depression scale was .89. For the present study, the other two sub-scales (i.e., anxiety and stress) were used as filler items.

#### Hikikomori risk factors

The Hikikomori Risk factors were measured by the NEET Hikikomori Risk Scale (NHR; Uchida & Norasakkunkit, 2015). The scale comprises 27 items covering various dimensions (e.g., social isolation, employment, and educational disengagement) related to Hikikomori risk factors. Each item (e.g., *Upon graduating school or college, I think that to work is to fulfil one’s duty to society*) is rated on a 5-point Likert Scale (1 = Strongly Disagree; 5 = Strongly Agree). The sum of the NHR is used to measure Hikikomori risk factors, with the higher NHR scores indicating a higher Hikikomori risk. The Cronbach’s α for the NHR was .85.

#### Hikikomori tendencies

The Hikikomori Questionnaire-25 (HQ-25; Teo et al., 2018) was used to measure Hikikomori tendencies. The HQ-25 comprises 25 items (e.g., *I stay away from other people*) with a 5-point Likert Scale (1 = Not at all; 5 = Extremely). The sum was used to measure severity of Hikikomori tendencies, with the higher scores indicating higher chance becoming Hikikomori. The Cronbach’s α for the HQ-25 was .96.

### Procedure

Participants were given a web link (Qualtrics) and where they provided consent to participate the study. Then, they were invited to answer a set of questionnaires that including (a) the demographic information, (b) technological addiction questionnaires (i.e., IAT, SAS-SV, IGDS9-SF), (c) depression (i.e., DASS-21), (d) Hikikomori risk factors (i.e., NHR), and (e) Hikikomori tendencies (i.e., HQ-25). The duration of the whole questionnaire was approximately 30 min. The study was approved by the University ethics board (H9355). The data for the present study is available at OSF, https://osf.io/p6auj/.

## Results

### Descriptives and correlations

The descriptives and the correlations between participant’s gender, technological addiction (i.e., IAT, SAS-SV, IGDS9-SF), depression, Hikikomori risk factors, and Hikikomori tendencies are presented in Table 1. Of note, the internet addiction (i.e., IAT), smartphone addiction (i.e., SAS-SV), and internet gaming disorder (IGD), were significantly positively correlated with Hikikomori risk factors (i.e., NHR) and Hikikomori tendencies (i.e., HQ-25).

**Table 1.** Descriptives and bivariate correlations between gender, technological addiction (internet addiction, smartphone addiction, internet gaming disorder), depression, hikikomori tendencies, and hikikomori risk factors.

	1. Gender	2. IAT	3. SAS-SV	4. IGD	5. Depression	6. NHR	7. HQ-25
1. Gender	–						
2. IAT	−.130	–					
3. SAS-SV	−.090	.661**	–				
4. IGD	−.258**	.622**	.373**	–			
5. Depression	.001	.448**	.341**	.319**	–		
6. NHR	−.116	.414**	.331**	.446**	.500**	–	
7. HQ-25	−.057	.406**	.295**	.427**	.473**	.695**	–
*M*	1.76	62.43	33.27	19.07	15.01	103.67	73.37
*SD*	0.51	20.30	10.98	8.09	5.56	20.86	17.10

*Note. N* = 182; Gender was coded as 1 = males; 2 = females, and 3 = others; IAT = Internet Addiction Test; SAS-SV = Smartphone Addiction Scale; IGD = Internet Gaming Disorder; Depression = DASS-21: Depression subscale; NHR = hikikomori risk factors; HQ25 = hikikomori tendencies.

* *p* < .05. **p < .01.

Given a gender skew (approximately 70.3% of the sample were females, we examined the relationship between gender and the other variables. Gender did not correlate with most of the variables (apart from IGD); we conducted a one-way between-subjects ANOVA to examine if gender has any impact on the outcome variable (i.e., Hikikomori tendencies). Results showed that the impact of gender on Hikikomori tendencies was not significant, *F*(3, 178) = 0.89, *p* = .45, η_p_^2^ = .02. Since gender did not affect Hikikomori tendencies, gender was not considered in the subsequent analysis.

### Examining the mediation models

With the correlation showing that technological addiction, depression, Hikikomori risk factors, and Hikikomori tendencies were significantly correlated, we examined the proposed mediational models. While some studies have suggested that Hikikomori risk factors could be part of the spectrum of Hikikomori tendencies, others have showed that Hikikomori risk factors could be an antecedent to Hikikomori tendencies (e.g., Lin et al., 2022). Hence, we conducted six separate serial mediation analysis by swapping the positions of technological addiction and depression at the predictor. Hikikomori risk factors were always served as the second mediating variable (MV2).

**Figure 2.**
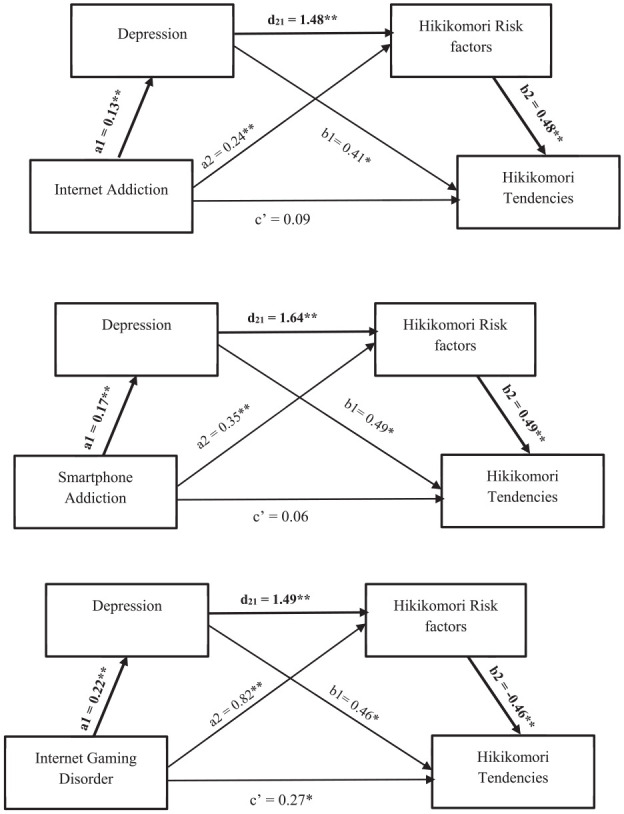
Pathways for sequential mediational analyses, with each aspect for technological addiction as the predictor. *Note*. In each path diagram, the coefficients a_1_ and a_2_ are the simple (zero-order) effects of the IV on MV_1_ and MV_2_, respectively. The coefficient d_21_ represents the sequential effect of MV_1_ on MV_2_, controlling for the IV, and coefficients b_1_, b_2_, and ć represent the partialled influences of MV_1_, MV_2_, and the IV on the DV when the IV and both mediators are used to predict the DV. Bold paths representing highest IE value in the model. *p < .05. **p < .01.

In Analysis 1 to 3, internet addiction, smartphone addiction, and internet gaming disorder were the respective predictors, depression was MV1, Hikikomori risk factors was MV2, and Hikikomori tendencies was the outcome variable. Results using PROCESS Macro Model 6 with 5,000 bootstrap re-sampling (Hayes, 2017) showed that depression (MV1) and Hikikomori risk factors (MV2) produced complete mediation when internet addiction and smartphone addiction were the predictors. When internet gaming disorder was the predictor, depression and Hikikomori risk factors produced partial mediation. All the indirect effects (IEs), in the 95% CIs bootstrap re-sampling excluding zero, were significant when depression served as MV1, and Hikikomori risk factors served as MV2, ranging from 0.11 to 0.19. Details of the beta weight are presented in Figure 2.

**Figure 3.**
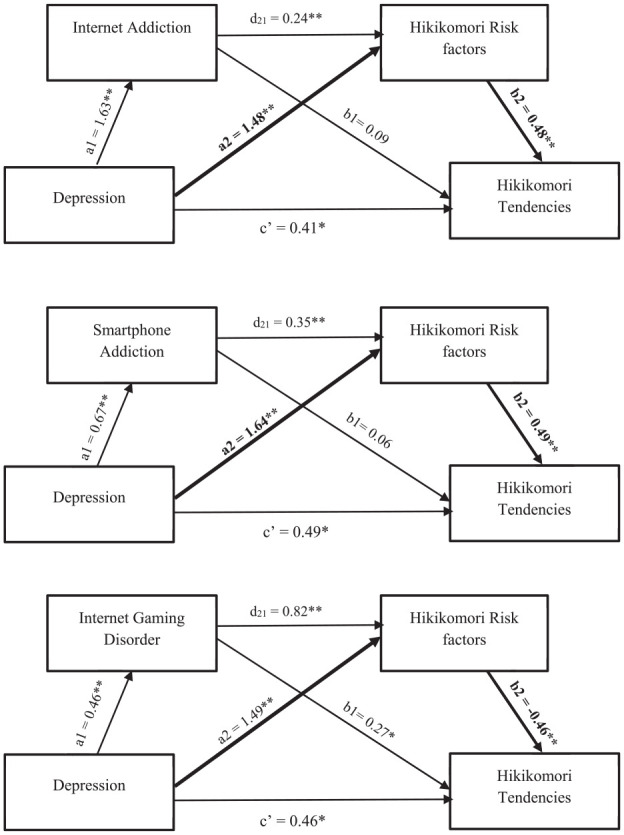
Pathways for Sequential Mediational Analyses, with Each Aspect for Technological Addiction as MV1. *Note*. Sequential Mediation Analysis 4, 5, and 6. In each path diagram, the coefficients a_1_ and a_2_ are the simple (zero-order) effects of the IV on MV_1_ and MV_2_, respectively. The coefficient d_21_ represents the sequential effect of MV_1_ on MV_2_, controlling for the IV, and coefficients b_1_, b_2_, and ć represent the partialled influences of MV_1_, MV_2_, and the IV on the DV when the IV and both mediators are used to predict the DV. Bold paths representing highest IE value in the model. *p = .05. **p < .01.

In Analyses 4, 5, and 6, we examined internet addiction, smartphone addiction, and internet gaming disorder respectively as MV1, Hikikomori risk factors as MV2, Hikikomori tendencies as the outcome variable, and depression as the predictor. Results from the PROCESS Macro Model 6 with 5,000 bootstrap re-sampling (Hayes, 2017) showed that depression was partially mediated by internet addiction, smartphone addiction, internet gaming disorder (MV1) and Hikikomori risk factors (MV2). The IEs ranged from 0.09 to 0.15. The IEs were stronger when the three technological addictions were removed from the mediational models. Details of the beta weights are presented in Figure 3.

## Discussion

Study 1 revealed four key findings. First, technological addiction (i.e., internet addiction, smartphone addiction, internet gaming disorder), depression, Hikikomori risk factors, and Hikikomori tendencies were positively correlated with each other. Second, gender did not influence Hikikomori tendencies. Third, technological addiction, depression, and Hikikomori risk factors were identified as potential contributing factors to Hikikomori tendencies. Fourth, when comparing two pathways – (1) technological addiction → depression → Hikikomori risk factors → Hikikomori tendencies versus (2) depression → technological addiction → Hikikomori risk factors → Hikikomori tendencies – the former demonstrated a stronger mediation effect (i.e., complete mediation and a stronger indirect effect). Therefore, it is reasonable to conclude that young adults addicted to SNS technologies are more likely to experience heightened depression, which in turn activates greater Hikikomori risk factors, ultimately leading to social withdrawal (i.e., higher Hikikomori tendencies).

The findings from Study 1 suggested that depression and Hikikomori risk factors were potential mediators in the relationship between technological addiction and Hikikomori tendencies. To test this hypothesis, Study 2 employed a quasi-experimental design to control for the direct impact of technological addiction on Hikikomori tendencies while measuring depression and Hikikomori risk factors.

## Study 2 – method

### Participants and design

We advertised Study 2 and highlighted that we were looking for SNS users via a university research participation pool and social media platforms (e.g., Telegram, WhatsApp). Power analysis suggested 102 participants would be needed in a two-group quasi-experimental design, with medium effect size (Cohen’s *d* = .50), power = .80, and alpha .05, one-tailed. A total of 141 undergraduates (*M* = 22.26, *SD* = 4.41; 39 males, 96 females, 6 others) were recruited for Study 2. Similar to Study 1, majority of participants were from Southeast Asia (71.38%). From the 141 undergraduates, 60 were considered high SNS users and 50 were considered low SNS users, resulting in a final sample of 110 participants. Details of dichotomizing high versus SNS users are provided in the Materials section.

In this quasi-experimental design, the independent variable (IV) SNS usage (high vs. low), and the measured variables (DVs) were depression, Hikikomori risk factors, and Hikikomori tendencies. The IV of SNS usage serves as a proxy for technological addiction as per Study 1.

### Materials

#### Social networking sites (SNS) usage

We recorded participant’s SNS usage by asking them three questions: (1) how much time (in hours) you spend on SNS for work per day, (2) how much time (in hours) you spend on SNS for socialization per day, and (3) how much time (in hours) you spend on SNS for entertainment per day. We summed the hours to obtain an overall SNS usage per day. Based on Internet Trend Reports (e.g., investment and internet companies), the average SNS usage is about 6 hr per day (Kidd, 2024; Leake, 2024). Research has indicated over 8 hr could be considered problematic SNS usage (e.g., Shannon et al., 2022; Yang et al., 2022). Hence, we considered participants who spent more than 8 hr as high SNS users and those who spend 6 hr and below would be considered as low SNS users. Those who spent 7 hr were removed from the analysis.

#### Depression, hikikomori risk factors, and hikikomori tendencies

The same measurements in Study 1 were used for Study 2. We administered scales for depression (DASS-21; Lovibond & Lovibond, 1995), Hikikomori risk factors (NHR; Uchida & Norasakkunkit, 2015), and Hikikomori tendencies (HQ-25; Teo et al., 2018).

### Procedure

All participants provided consent to participate the study, after they entered the Qualtrics link. The same set of (a) the demographic information, (b) depression (i.e., DASS-21), Hikikomori risk factor (i.e., NHR), and Hikikomori tendencies (i.e., HQ-25) questionnaires were administered. The SNS usage questions were included as part of the demographic information. The duration of the whole questionnaire was approximately 20 min. The study was approved by the University ethics board (H9355 ver. 2). Data for the present study can be accessed via https://osf.io/p6auj/.

## Results

### Descriptives, correlations, and ANOVA

Correlational coefficients showed that SNS Usage, depression, Hikikomori risk factors, and Hikikomori Tendencies were positively related with each other. The descriptives and the correlations between SNS usage, depression, Hikikomori risk factor, and Hikikomori tendencies were presented in Table 2.

**Table 2.** Descriptives and bivariate correlations between SNS usage, depression, hikikomori risk factors, and hikikomori tendencies.

	1. SNS Usage	2. Depression	3. NHR	4. HQ-25
1. SNS Usage	–			
2. Depression	.390**	–		
3. NHR	.270**	.561**	–	
4. HQ-25	.212**	.489**	.639**	–
*M*	0.55	28.25	72.04	105.05
*SD*	0.50	9.81	16.61	18.27

*Note. N* = 110; SNS usage was coded as 1 = high users; 2 = low users; Depression = DASS-21: Depression subscale; NHR = hikikomori risk factors; HQ25 = hikikomori tendencies.

*p < .05. **p < .01.

We continued with three separate independent t-tests to examine if SNS usage would impact depression, Hikikomori risk factors, and Hikikomori tendencies. Results showed that there high SNS users (*M* = 31.73, *SD* = 8.77) were more depressed compared to low SNS users (*M* = 24.08, *SD* = 9.42), *t*(108) = 4.41, *p* < .001, Cohen’s *d* = 0.84; (b) high SNS users (*M* = 108.56, *SD* = 15.37) scored higher on Hikikomori risk factors compared to low SNS users (*M* = 100.83, *SD* = 20.62), *t*(108) = 2.25, *p* = .013, Cohen’s *d* = 0.43; and (c) high SNS users (*M* = 76.12, *SD* = 15.72) were more prone to Hikikomori tendencies compared to low SNS users (*M* = 67.14, *SD* = 16.47), *t*(108) = 2.92, *p* = .002, Cohen’s *d* = 0.56. Overall, high SNS users resulted in higher levels in depression, Hikikomori risk Factors, and Hikikomori Tendencies, compared to low SNS users.

### Serial mediation analysis

**Figure 4.**
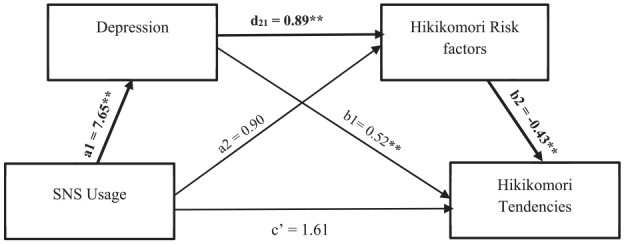
Serial mediation analysis with SNS usage as predictor, depression as MV1, Hikikomori risk factors as MV2, and Hikikomori tendencies as the outcome. Note. The coefficients a_1_ and a_2_ are the simple (zero-order) effects of the IV on MV_1_ and MV_2_, respectively. The coefficient d_21_ represents the sequential effect of MV_1_ on MV_2_, controlling for the IV, and coefficients b_1_, b_2_, and ć represent the partialled influences of MV_1_, MV_2_, and the IV on the DV when the IV and both mediators are used to predict the DV. Bold paths representing significant IE in the model. *p = .05. **p < .01.

Following the results in Study 1, we conducted a serial mediation analysis with SNS usage as the predictor, depression as MV1, Hikikomori risk factors as MV2, and Hikikomori tendencies as the outcome variable. Results from PROCESS Macro Model 6 with 5,000 bootstrap re-sampling (Hayes, 2017) showed that depression (MV1) and Hikikomori risk factors (MV2) produced complete mediation. The indirect effect (IE) was 2.97, the 95% CIs bootstrap re-sampling excluding zero^1^. Details of the beta weights are presented in Figure 4.

## Discussion

In Study 2, we conducted a quasi-experiment by comparing high and low SNS users on depression, Hikikomori risk factors, and Hikikomori tendencies. Additionally, we investigated if depression and Hikikomori risk factors mediate the relationship between SNS usage and Hikikomori tendencies. The results showed that (a) high SNS users were more depressed, exhibited higher Hikikomori risk factors, and were more prone to becoming Hikikomoris, and (b) the pathway from SNS usage (i.e., technological addiction) → depression → Hikikomori risk factors → Hikikomori tendencies was significant, indicating complete mediation.

Hence, the results suggest that depression precedes Hikikomori risk factors in the link between SNS usage (i.e., technological addiction) and Hikikomori tendencies. Simply put, young adults who are high SNS users may become more depressed, leading to increased Hikikomori risk factors (e.g., refusing to work or attend school), which, in turn, result in social withdrawal (i.e., higher Hikikomori tendencies).

## General discussion

### Key findings

The present research investigated the relationships between technological addiction, depression, Hikikomori risk factors, and Hikikomori tendencies among the young adults. Five key findings emerged across the two studies. First, technological addiction, depression, and Hikikomori risk factors were positively correlated with Hikikomori tendencies. Second, no significant gender differences were observed in Hikikomori tendencies. Third, depression and Hikikomori risk factors were identified as potential mediators between technological addiction and Hikikomori tendencies. Fourth, we proposed two possible mediational pathways systematically and found that when technological addiction (i.e., internet, smartphone, online gaming addiction) was the predictor, there was a stronger indirect effect via Hikikomori risk factors to Hikikomori tendencies, compared to when depression was the predictor. Fifth, in the quasi-experiment, high SNS users exhibited greater levels of depression, higher Hikikomori risk factors, and stronger Hikikomori tendencies compared to low SNS users. Consistent with findings from Study 1, Study 2 also revealed that depression and Hikikomori risk factors completely mediated the relationship between SNS usage and Hikikomori tendencies.

Previous research has shown that technological addiction, depression, hikikomori risk factors, and hikikomori tendencies are interrelated, with bidirectional relationships observed across these variables (e.g., Amendola et al., 2023; Tang & Koh, 2017; Xie et al., 2023). Our findings aligned with these earlier studies. Specifically, when examining subtypes of technological addictions – internet, smartphone, and online gaming – all three demonstrated positive correlations with both hikikomori risk factors and withdrawal tendencies. Interestingly, gender was not associated with internet addiction, smartphone addiction, Hikikomori risk factors, or Hikikomori tendencies, but it was linked to online gaming addiction. Furthermore, we found that gender had no influence on Hikikomori tendencies. Although previous studies (e.g., Frankova, 2019; Liew et al., 2021; Lin et al., 2022) have suggested that demographic and psychosocial factors (e.g., income, school refusal) may affect Hikikomori tendencies, our study did not reveal any significant differences in Hikikomori tendencies between male and female young adults.

A central question in Hikikomori research involves understanding directional causality. Our mediational analyses demonstrated that the pathway of technological addiction → depression → Hikikomori risk factors → Hikikomori tendencies exhibited complete mediation, with a stronger indirect effect compared to the other pathway (depression → technological addiction → Hikikomori risk factors → Hikikomori tendencies). While prior research suggests that depressed young adults may develop technological addiction, engage in Hikikomori risk factors (e.g., school absenteeism), and eventually adopt Hikikomori behaviors (e.g., Shah et al., 2024), our findings propose an alternative: young adults with technological addiction may experience exacerbated depressive symptoms. This, in turn, increases engagement in Hikikomori risk factors and heightens Hikikomori tendencies. Furthermore, our mediational analyses indicated that depression and Hikikomori risk factors could act as sequential mediators between technological addiction and Hikikomori tendencies.

When experimentally examining technological addiction as a quasi-variable, we found that high SNS users exhibited elevated depression levels, engaged in more Hikikomori risk factors, and demonstrated stronger Hikikomori tendencies. Across both the cross-sectional and quasi-experimental studies, technological addictions – including internet, smartphone, online gaming, and SNS usage – emerged as the primary predictive variable. Extant research postulates that pandemic-related societal shifts (e.g., Vahedi & Zannella, 2021) intensified technology reliance among adolescents and young adults. Hence, the addictive patterns and withdrawal effects from technological use could exacerbate depressive symptoms (e.g., Lee et al., 2013; Tang & Koh, 2017). As young adults experience heightened depression, they may begin avoiding school or work (i.e., Hikikomori risk factors), ultimately leading to Hikikomori tendencies.

### Limitations and future directions

While our research identified similar correlations between gender and online gaming addiction as other studies (e.g., Tateno et al., 2019), it did not find a significant association with hikikomori tendencies. One potential reason for the non-significance could be due to the imbalanced gender distribution in our sample. This gender imbalance is not unique to our study, as several other Hikikomori studies have also reported skewed gender ratios (e.g., Liew et al., 2021). Although males are often reported to exhibit Hikikomori behaviors more frequently due to societal expectations (e.g., Kanai et al., 2024; Malagón-Amor et al., 2015), emerging evidence suggests these tendencies may also manifest in females, with potential underreporting of female cases (Yong, 2024). Additionally, since our sample predominantly comprised participants from Southeast Asian countries (69%–71%), future studies should prioritize balanced gender and geographical representation to enhance generalizability.

Another caveat of the present research lies in its design. Although the paired-study incorporated a quasi-experimental component (Study 2) to complement the correlational design (Study 1), the overall nature of the research remains cross-sectional. While we proposed the mediational role of depression and Hikikomori risk factors in the link between technological addiction and Hikikomori tendencies, it is important to clarify that this study does not claim to establish a definitive causal relationship between these variables. The aim of our research was exploratory in nature, and the proposed mediational/directional model requires further robust theoretical and empirical support. Therefore, future researchers are encouraged to investigate alternative mediational pathways using interrupted time-series designs or controlled longitudinal approaches to strengthen causal inferences (e.g., Koyama et al., 2010).

In the same vein, due to the lack of theoretical frameworks and existing models explaining the causal relationship between technological addiction and Hikikomori tendencies, the present paired-study was limited to exploring two plausible (though rational) models. Consequently, this approach may have overlooked critical variables for alternative models. Past research highlights potential contributors such as psychological needs (e.g., Katsuki et al., 2019), self-concept (e.g., Norasakkunkit, 2014), self-esteem (e.g., Liew et al., 2021), relational mobility (e.g., Yuki & Schug, 2020), and academic performance (Skues et al., 2016). Studies also suggest comorbidity between variables may exacerbate Hikikomori tendencies – for instance, excessive Facebook use (a form of internet addiction) negatively impacts GPA, potentially leading to school refusal and Hikikomori behaviors (e.g., Benarous et al., 2022; Rosen et al., 2013; Ryan et al., 2014; Zhang et al., 2022). Future research should investigate whether these variables act as predictors, mediators, or moderators, in Hikikomori development.

## Conclusion

This study examined the relationships among technological addiction, depression, Hikikomori risk factors, and Hikikomori tendencies, finding significant positive correlations between all variables. Subsequently, two potential pathways were systematically tested. The pathway of ‘technological addiction → depression → Hikikomori risk factors → Hikikomori tendencies’ demonstrated a more coherent progression compared to ‘depression → technological addiction → Hikikomori risk factors → Hikikomori tendencies’. Based on these findings, a quasi-experiment was conducted, revealing that young adults with high social networking site (SNS) usage exhibited greater depression, elevated Hikikomori risk factors, and stronger Hikikomori tendencies.These results also support the findings from Study 1, which indicated that depression and Hikikomori risk factors serially mediate the relationship between SNS usage and Hikikomori tendencies.

In sum, the present study suggests that technological addiction may contribute to depression among young adults. In a depressive state, individuals are more likely to engage in Hikikomori-related risk behaviors, such as avoiding school or work, which could ultimately result in becoming Hikikomoris. To mitigate this issue, it is recommended that organizations and government agencies implement measures to reduce excessive technology use and address depressive symptoms in young adults to prevent the development of Hikikomori tendencies (Kato et al., 2024; Wong et al., 2019).

## Notes

1. With depression → technological addiction → Hikikomori risk factors → Hikikomori tendencies pathway, the IE was 0.92 and marginally significant, hence, the results were not indicated in the Results section.
